# Appraising Adjuvant Endocrine Therapy in Hormone Receptor Positive HER2-Negative Breast Cancer—A Literature Review

**DOI:** 10.3390/curroncol29070394

**Published:** 2022-07-13

**Authors:** Danilo Giffoni de Mello Morais Mata, Carlos Amir Carmona, Andrea Eisen, Maureen Trudeau

**Affiliations:** 1 Odette Cancer Centre, Sunnybrook Health Sciences Centre, Toronto, ON M4N3M5, Canada; carlos.carmona@sunnybrook.ca (C.A.C.); andrea.eisen@sunnybrook.ca (A.E.); maureen.trudeau@sunnybrook.ca (M.T.); 2 Department of Medicine, Division of Medical Oncology and Hematology, University of Toronto, Toronto, ON M5S1A1, Canada

**Keywords:** early breast cancer, premenopausal, postmenopausal, hormone receptor positive, HER2-negative, endocrine therapy, selective estrogen receptor, aromatase inhibitors, adjuvant cyclin-dependent kinases 4/6 inhibitors, bisphosphonates

## Abstract

Background: Approximately 75% of breast cancer (BC) is associated with luminal differentiation expressing endocrine receptors (ER). For ER+ HER2− tumors, adjuvant endocrine therapy (ET) is the cornerstone treatment. Although relapse events steadily continue, the ET benefits translate to dramatically lengthen life expectancy with bearable side-effects. This review of ER+ HER2− female BC outlines suitable adjuvant treatment strategies to help guide clinical decision making around appropriate therapy. Methods: A literature search was conducted in Embase, Medline, and the Cochrane Libraries, using ER+ HER−, ET BC keywords. Results: In low-risk patients: five years of ET is the standard option. While Tamoxifen remains the preferred selection for premenopausal women, AI is the choice for postmenopausal patients. In the high-risk category: ET plus/minus OFS with two years of Abemaciclib is recommended. Although extended ET for a total of ten years is an alternative, the optimal AI duration is undetermined; nevertheless an additional two to three years beyond the initial five years may be sufficient. In this postmenopausal group, bisphosphonate is endorsed. Conclusions: Classifying the risk category assists in deciding the treatment route and its optimal duration. Tailoring the breadth of ET hinges on a wide array of factors to be appraised for each individualized case, including weighing its benefits and harms.

## 1. Background

Among females, breast cancer (BC) is the fifth leading cause of cancer-related death worldwide, contributing to almost 12% of all cancer cases [1]. Approximately 75% of BC is associated with luminal differentiation expressing endocrine receptors (ER) [2]. Harboring ER expression is a predictive factor for endocrine therapy (ET) response and has a promising survival outcome with a dramatic risk reduction in local and distant metastases [3,4]. In contrast, this group typically demonstrates an insufficient chemotherapy response [5]. To ascertain for which patients the magnitude of the adjuvant chemotherapy effect will not be suitable, genomic expression assays help to predict the risk of cancer recurrence and identify those for which ET alone is advantageous [6,7,8,9]. ET is distinctly efficacious among the luminal tumors. Regardless of the following factors that affect the ET response, including the level of ER positivity and tumor-infiltrating lymphocyte, cancer morphology, or germline mutation carriage, they are frequently treated as a singular entity [10,11,12]. Interestingly, even within the highly ER positive group, BRCA2 carriers are predictive of poor ET effectiveness [12,13]. Similarly, a diverse response to ET is seen between the pure ductal and lobular carcinomas versus mixed or hybrid histology [10,14].

Approximately one out of six women with ER+ and human epidermal growth factor receptor 2 (HER2) negative, with a malignant affected lymph node (LN), will have disease relapse reflecting the high association between LN status and the rates of BC recurrence and mortality [4,15]. This is compounded by the importance of adequate treatment adherence, as compliance is highly correlated with better outcomes [3]. 

There are four BC subtypes: ER+ HER2−, ER+ HER2+, ER− HER2+, and triple negative breast cancer, characterized by ER− HER2− [16]. This review of ER+ HER2− female BC will outline suitable treatment strategies in the adjuvant or postoperative setting to help guide clinical decision making around appropriate therapy.

This article highlights the latest evidence of ET and its optimal treatment duration, including aromatase inhibitors (AIs), selective estrogen receptor modulators (SERMs), and ovarian function suppressors (OFS), as well as the usage of the novel cyclin-dependent kinase four and six (CDK4/6) inhibitors and bisphosphonates (Table 1).

## 2. Materials and Methods 

A literature search for clinical trials, systematic reviews and meta-analyses was conducted by searching Embase, Medline, and the Cochrane Library. The search targeted published medical articles in the English language between January 2012 and April 2022. Literature was included from scientific journals, as well as abstracts from oncology conferences, to ensure inclusion of recent medical-based evidence relevant to clinical practice. 

## 3. Endocrine Status and Adjuvant Endocrine Therapy

Immunohistochemistry (IHC) is an essential assay to determine the expression of endocrine subtype profiling [29]. For treatment-making decisions, the challenge lies around determining the ER expression cut off at which patients will benefit from ET. In ER-low positive tumors (1–10% of IHC+) which comprise up to 3% of BC patients, ET is not advantageous [3,29,30]. This is attributed to the heterogeneity of the tumor pathogenesis being more similar to the basal-like, rather than the luminal phenotype [29]. With respect to the progesterone receptor (PR) status, for tumors that are ER+, the PR is not predictive of ET efficacy [3].

The role of adjuvant ET is to eradicate potential undetected micrometastatic ER-enriched tumor cells. Evaluating factors such as patient preference, menopausal status, and medical history, as well as pathological tumor features, are decisive to guiding treating physicians towards the breadth of ET selection for each individual case [3]. Determining the risk category helps determine the treatment duration [4].

## 4. Menopausal Status 

Premenopausal women contribute to approximately one third of all BC cases [28,31]. In this population, the main ovarian hormone secreted is 17β-estradiol [2,32]. In the microenvironment of the breast epithelium and mammary gland, endogenous hormone signaling is mediated by estrogen and progesterone receptors. Through DNA transcription factors, the physiological sex steroidal activity can stimulate stem cells to an eventual development of endocrine enhanced tumors [2,33].

Within the SERM class, Tamoxifen was a pioneer for ET in BC, and data around its use extends over four decades [34]. Numerous other SERMs have been studied, such as Raloxifene, Toremifene, and Endoxifen, but to date, the benefit of Tamoxifen remains unsurpassed within this class of medications [34,35]. By competitive mechanisms of binding to ERs, Tamoxifen can drive contrasting endogenous activity depending on the targeted cell. Its inhibitory effect on estrogen-regulated pathways leads to suppression of mammary tumor angiogenesis. In addition, as an estrogen agonist, Tamoxifen has a cardioprotective effect, but conversely has an increased risk of venous thromboembolism as well as hyperplasia or tumorigenesis in the endometrium [36,37]. 

**Figure 1:**
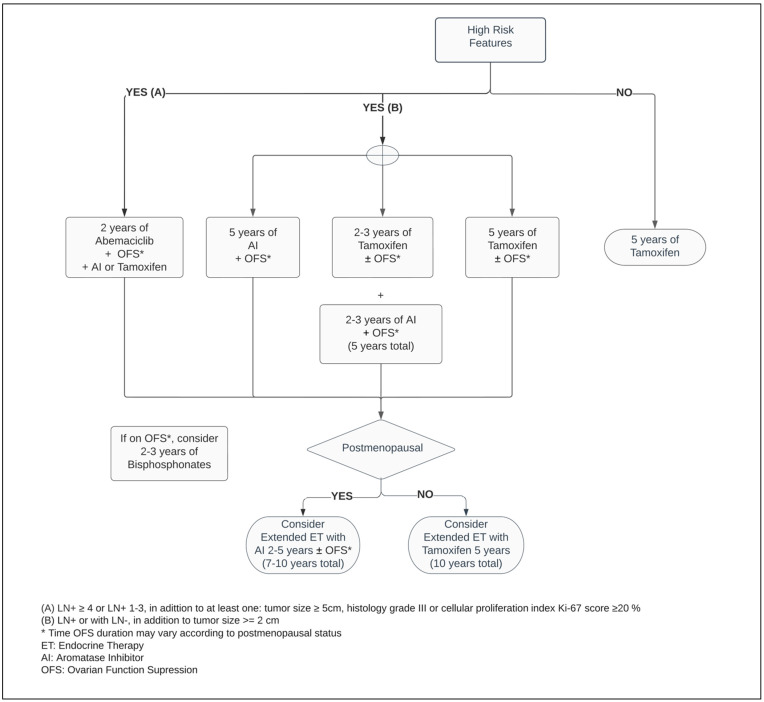
Adjuvant endocrine therapy for premenopausal women with ER+ HER2− breast cancer.

Regardless of the menopausal status, Tamoxifen is a suitable adjuvant therapy, and continues as the main ET option for premenopausal women with ER+ BC (Figure 1) [3,38,39]. Five years of Tamoxifen therapy can reduce the risk of recurrence by approximately 40% and decrease mortality by a third when compared with no ET, with a carryover benefit extending beyond ten years [3].

In postmenopausal women, the main source of estrogen comes from extragonadal tissues and is mediated by aromatase, a crucial enzyme responsible for a cascade of steroid synthesis and regulation. The AIs substantially reduce the circulating estrogen within plasma levels by suppressing its conversion from androgens, predominantly in adipose tissues. Hence, it leads to vasomotor symptoms such as hot flashes and vaginal dryness, as well as arthralgia, lipid metabolism dysregulation and bone mineral loss [37,40].

**Figure 2:**
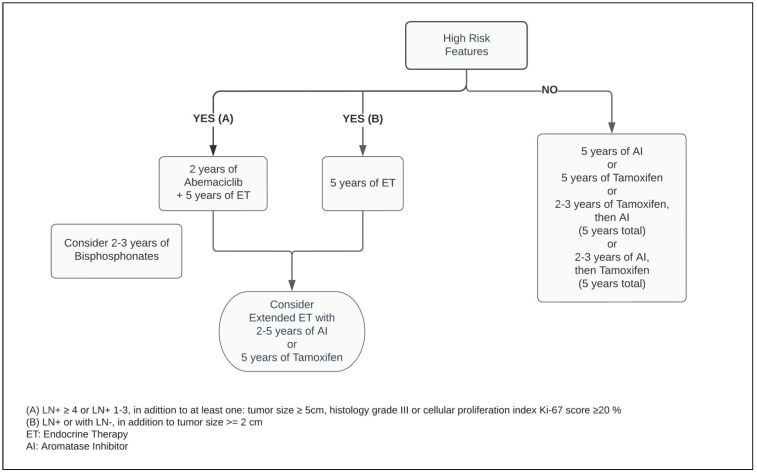
Adjuvant endocrine therapy for postmenopausal women with ER+ HER2− breast cancer.

Five years of adjuvant treatment with AI in postmenopausal women has a similar efficacy and safety profile among Anastrozole, Letrozole and Exemestane [41,42,43]. When compared with Tamoxifen, the AIs have shown to be superior in postmenopausal patients, reducing the risk of mortality by approximately 15% and distant and local recurrence by 14% and 26%, respectively, at ten years (Figure 2) [44].

For perimenopausal patients with pathological low-risk characteristics, an alternative option is a five year ET regimen, consisting of Tamoxifen upfront, followed sequentially by AI [44,45]. Although the most substantial recurrence risk reduction from switching therapy occurs within the total five year time on treatment, the benefit of exposing patients to AI for two to three years decreases mortality related to breast cancer by 16% at one decade, compared with being on Tamoxifen alone for five years [44]. 

## 5. Classification of High-Risk

There is extensive evidence around LN+ being predictive of lower survival outcomes. In patients with a number of axillary LN+ 1–3 and LN+ ≥ 4, the mortality risk from BC at twenty years from initial diagnosis is 28% and 48%, respectively, with an absolute increased risk for death by at least 13%, when compared with LN 0 [4].

The definition of adverse clinic/pathologic features for BC recurrence varies. Within the American Society of Clinical Oncology (ASCO) guidelines, two categories of high-risk have been established based on prognostic characteristics and the likelihood rates of disease relapse [46,47]. These stipulate different high-risk thresholds, taking into consideration the benefit of therapeutic options for distinct scenarios: firstly, for any BC patient with LN+ or with LN−, in addition to tumor size ≥ 2 cm, the recommendations are to use extended endocrine therapy and/or an adjunct of ET with OFS, the latter combination being exclusively endorsed in premenopausal women. The next scenario, which involves patients with LN+ ≥ 4 or LN+ 1–3, in addition to at least one of the following characteristics—tumor size ≥ 5 cm, histology grade III, or cellular proliferation index Ki-67 ≥ 20%—fits the indication for adjuvant ET in combination with two years of Abemaciclib [47].

Moreover, there is an uncertainty about whether amongst those with LN- and tumor size ≥ 1 cm, with adverse histological grade and/or elevated recurrence score in genomic profiling assays, are also deemed to fall within a high-risk classification [46].

## 6. Ovarian Function Suppressors (OFS)

Definitive and effective transitory methods can be employed to decrease the production of sex hormones to postmenopausal range values. The first consists of a bilateral oophorectomy or directed radiation to the ovaries [48,49]. The second is through a transient drug effect induced by OFS such as the luteinizing hormone (LH)-releasing hormone (LHRH) analogs [50]. As an initial effect of chemical castration, the serum estradiol and progesterone levels are increased. Its regular administration promotes downstream inhibitory cascades in the hypothalamic–pituitary axis to the gonadotropic hormones, decreasing the secretion of the follicle stimulating hormone (FSH) and LH, hence suppressing the gonadal estrogen levels [21,22,50,51].

A high-certainty evidence-based systematic review which included studies such as SOFT and TEXT, comprised more than eleven thousand premenopausal patients. Thereby, it demonstrated that regardless of the ET of choice for premenopausal BC, the addition of OFS agents, administered monthly to adjuvant ET, reduced the risk of mortality by 14%, as well as disease-free survival (DFS) and contralateral BC by 17% and 25%, respectively, when compared with ET alone. While the adjunct administration of OFS between one and three years resulted in a mortality reduction, its prolonged use for over three years enhanced the DFS endpoint. However, there is insufficient randomized data evidence around OFS in the extended adjuvant setting beyond five years. In patients who did not receive chemotherapy, combining OFS to ET did not improve survival or decrease recurrence rates [52]. Considering previous exposure to chemotherapy as an acceptable surrogate from which an overall risk assessment demonstrates a higher risk for cancer recurrence, this suggests that only a select group of patients may benefit from OFS in the adjuvant setting. This inference is reinforced by the pathologic feature of LN involvement being a predictive factor for a superior efficacy of the ET with OFS, significantly improving OS and DFS outcomes (Figure 1) [52,53,54].

There are contradicting data regarding survival outcomes between Tamoxifen and AI in combination with OFS. A recent patient-level meta-analysis developed by the Early Breast Cancer Trialists’ Collaborative Group (EBCTCG) included more than seven thousand BC participants enrolled in randomized controlled trials (RCTs). The study results did not reveal an OS difference between Tamoxifen and various AIs, even though both arms utilized OFS as adjunct therapy. However, the latter demonstrated a lower local and distant BC recurrence rate by at least 20%, when compared with Tamoxifen, with an absolute risk reduction of approximately 3% in five and ten years. No apparent benefit was seen between these two classes of ET with regard to the subgroups of patients with HER2+ or affected with LN ≥ 4 [55]. 

Initiating OFS increases vasomotor symptoms, such as hot flashes and vaginal dryness and it may be a risk factor for osteoporosis. Therefore, there is a need to weigh the benefits and risks for each customized treatment option [52].

## 7. Extended Endocrine Therapy (EET)

There is a wide range of variation regarding survival endpoint achievement in RCTs with EET beyond the standard five year duration [4,56]. However, the consensus is that EET should be dedicated to patients harboring pathologically high long-term risks for a total duration of no longer than ten years [46,57].

The ATLAS study revealed that continuing Tamoxifen to ten years, versus concluding at five years, notably extends OS and DFS, with an absolute risk reduction for BC recurrence and mortality by 3.7% and 2.8%, at five years after the extended therapy was completed [58]. Nevertheless, it elevates the absolute cumulative risk to develop endometrial cancer by 1.7% [59]. Irrespective of whether the initial five years on ET used Tamoxifen, AI, or both (sequential switching therapy), EET with AI for an additional two to five years improves the risk of DFS by approximately 23% in high-risk postmenopausal women. Unfortunately, compilations of RCTs in a high-level evidence systematic review and meta-analysis did not demonstrate OS with an extended duration of AI therapy [40]. On the contrary, its prolonged exposure significantly increases musculoskeletal pain and increases the risk for cardiovascular events, fractures, and osteoporosis [40,60,61]. 

Interestingly, while in the five year ET duration, the PR status was irrelevant to predict ET response in patients with tumors ER overexpressed, in the EET setting, harboring double endocrine receptors which significantly enhance the magnitude of the ET effect when compared with BC carrying a single positive biomarker [3,62].

It is essential to identify the subgroup of patients in whom EET would be beneficial and outweigh its potential impact on quality of life due to treatment side effects, taking into consideration that its largest benefit occurs during the second decade after treatment and in high-risk patients [4,40,63]. Within the postmenopausal group, irrespective of the type of prior ET, patients should be considered for EET with AI for an additional five years [46]. However, the optimal duration of extending AI beyond five years remains imprecise, but in this context, an exposure of two to three years may be sufficient to prevent contralateral and recurrent events of BC (Figure 2) [46,64].

## 8. Genomic Expression Assays (GEAs)

Through evaluation of tumor biology using reference molecular drivers of cancer-related genes, GEAs generate prognostic information to estimate recurrence rates in the ER+ HER2- early BC [6,7]. Producing a grading risk score, GEAs identify those for whom adjuvant chemotherapy is not advantageous. Albeit, for those in whom the magnitude of effect of chemotherapy is not substantial, ET has a paramount role [6,7,8,9]. 

There is paucity of evidence that genomic profiling can determine adjuvant ET duration [9]. However, emerging studies have started to portray support in this area. Among the GEAs, the Breast Cancer Index (BCI) appraises the ratio of estrogen signaling and tumor proliferation, and thereby predicts ET efficacy [6,65]. Regardless of whether the ET class administered in the EET setting is the same as the primary adjuvant therapy, BCI has demonstrated to be a prognostic and predictive tool suitable for identifying patients in whom EET is beneficial [65,66,67]. The Clinical Treatment Score post-5 years (CTS5) is an online algorithm-based predictor tool that uses clinical and pathological features to calculate the ratio for late distant BC relapse after five years of adjuvant ET completion [9,68].

Although the BCI and the CTS5 have a moderate evidence-based strength, the most recent ASCO guideline endorses that either tool should be considered to support decision-makers towards the use of EET in patients treated with five years of adjuvant ET. The recommended groups for which there is excellent predictive benefit and where BCI should be applied, are patients with LN 0 or LN+ 1-3. CTS5 should be considered for postmenopausal patients only [9].

## 9. Adjuvant CDK4/6 Inhibitors and Endocrine Therapy

Critical measures to advance novel therapies are necessary for improving treatment outcomes in the high-risk groups. The successful results of CDK4/6 inhibitors in advanced BC patients prompted the emergence of new studies extending this drug class to non-metastatic scenarios [69,70,71,72,73]. The Pallas and Penelope-B trials combined Palbociclib with ET in adjuvant and neoadjuvant BC populations, respectively. While the first study investigated Palbociclib for two years, the latter planned its administration for one year only. Markedly, both studies failed to demonstrate their survival- and efficacy-related endpoints of adding Palbociclib to ET [74,75,76].

Meanwhile, the MonarchE trial, combining ET with two years of Abemaciclib in the postoperative setting in patients with adverse pathological LN+ presentation, decreased the risk of local–regional and distant recurrence by at least 25% when compared with ET alone [77]. Regardless of the index Ki-67, the absolute benefit of adding Abemaciclib to improving the risk of BC relapse reached 5.4% at 3 years [73,78]. As a response to these outstanding results, the ASCO guideline optimized recommendation of Abemaciclib plus ET to patients categorized within the high-risk group (Figure 1 and Figure 2) [47]. Notably, independent of the menopausal status, either Tamoxifen or AI plus or minus OFS (if applicable) were used in the MonarchE study [77].

The discrepant results between Palbociclib and Abemaciclib in adjuvant trials could be due to a substantially large premature discontinuation of Palbociclib and a broader population heterogeneity, including a wider range of cancer staging in the Pallas study. However, no benefit of Palbociclib was seen in the subgroup analysis of high-risk patients, nor in the comparison between those who discontinued, versus completed, the two years of the treatment regimen [74,75].

The disease progression of ER+ tumors follow an indolent pattern, and it may take years to reach robust data of death events, justifying why the OS data are immature in all CDK4/6 studies in the adjuvant setting [79]. Furthermore, there are large expectations about the upcoming preliminary results of the utilization of Ribociclib in early BC from the Natalee trial. This will help clarify the differing results of the outlined studies.

## 10. Bone-Modifying Agents (BMAs)

Bisphosphonates act by inhibiting osteoclasts by way of apoptosis, and thereby decrease bone resorption and increase mineralization [80,81]. Independent of the ER and HER2 status, the usage of bisphosphonates improves OS and DFS, and lower rates of bone metastasis in adjuvant breast cancer [82]. These effects are restricted to postmenopausal women and a higher magnitude of treatment effects may be encountered in those with an elevated risk for BC recurrence [83]. In this group, the time-to-event outcome showed a reduction in risk of mortality by 23% and disease recurrence by 18% when compared with no BMAs. Nevertheless, employing adjuvant bisphosphonates has a protective factor by reducing the risk of bone fracture events by more than 25%. Although adjuvant Denosumab was advantageous in minimizing skeletal events such as bone metastasis and fractures, it did not fulfill survival endpoints [82]. Although, upcoming OS results on the ABCSG-18 trial are expected shortly [84]. 

Cancer Care Ontario, in conjunction with ASCO, recommend one of following bisphosphonate agents: oral clodronate, oral ibandronate, or intravenous zoledronic acid (Figure 1 and Figure 2). An early start, within two-to-three months from the end of adjuvant chemotherapy or curative-intent surgery, leads to better BMAs efficacy [83]. Its usage is not exempt from side effects, such as bone pain, fatigue, potential rare episodes of hypocalcaemia, and osteonecrosis of the jaw, and should be disclosed to patients [82,83]. The latter encompasses 0.7% of cases and it has an increased likelihood with invasive dental surgical procedures [82,83,85]. Although within the uncommon range of adverse events, while the intravenous BMAs can potentially cause infusion reactions, nephrotoxicity, and ocular inflammation, the oral agents are more prone to gastrointestinal side effects [86].

In each specific case, decisions should be made by identifying the eligible patients that would benefit most from adjuvant BMAs, as those within the low-risk categories may lack meaningful treatment advantage. Other factors such as the patient’s preferred mode of administration, and comorbidity history, may influence clinicians’ decision-making around the adjuvant BMAs’ options [83].

## 11. Conclusions

Breast tumors associated with luminal differentiation ER+ HER2−, comprise the largest subgroup of female BC. In the adjuvant setting, its cornerstone treatment relies on ET, and its benefits translate dramatically to lengthen life expectancy with bearable side-effects. Nonetheless, relapse events steadily continue beyond the time of treatment completion, regardless of ET duration [4]. Tailoring the breadth of endocrine therapies hinges on a wide array of factors to be appraised by the prescribing physician, such as the patient’s menopausal status and the pathological tumor landscape. Classifying the risk category for the BC assists in deciding the treatment route and its optimal duration. In a select group of patients, GEAs predict those for whom chemotherapy is not beneficial and thereby for whom ET is the preferred choice. A meticulous disclosure of each suitable ET helps clinicians and patients to choose the appropriate therapy for each individualized case, outweighing its benefit and conceivable harm. Additionally, emphasizing an adequate treatment adherence is a crucial factor in contributing to satisfactory outcomes.

Furthermore, elderly patients are commonly underrepresented in randomized controlled studies. Hence, a thorough collection of medical history and special attention is required with respect to potential detrimental drug interaction in this population, and any added medicine should be cautiously selected.

This literature review highlights the latest evidence of ET in ER+ HER2− BC (Table 2). In summary, in patients whose risk of BC recurrence is low, five years of adjuvant ET is indicated as the standard of therapy. While Tamoxifen remains the preferred therapeutic option for premenopausal women, AIs are the drug of choice in the postmenopausal group. Moreover, the course of AI during the entire treatment duration, or its exposure for at least two years out of the five-year interval, provides a slightly superior DFS outcome. In those within the high-risk category, regardless of whether the endocrine ovarian function remains physiologically active, the most updated recommendation is upfront ET plus or minus OFS (if appropriate), in combination with two years of Abemaciclib. 

Following this risk classification, the addition of OFS to ET is advantageous in premenopausal patients, again with a discrete recurrence rate benefit favoring the AIs. The EET beyond the standard duration Tamoxifen or AI, up to ten years, is recommended, although the precise duration for the latter treatment after five years on AI is undetermined. Subsequently in this group, an additional two to three years of AI in the EET setting may be sufficient. Within postmenopausal high-risk patients, an early start of adjuvant bisphosphonates is endorsed.

## Figures and Tables

**Table 1:** Recommended Adjuvant Therapy for Women with ER+ HER2- Breast Cancer.

Drug Class	Medication	Dose/Administration	Mechanism of Action
SERM	Tamoxifen [17]	20 mg PO once daily	Inhibitory effect on estrogen-regulated pathways through competitive mechanisms of ER-binding, leading to suppression of mammary tumor angiogenesis
AI Non-Steroidal Steroidal	Anastrozole [18] Letrozole [19] Exemestane [20]	1 mg PO once daily 2.5 mg PO once daily 25 mg PO once daily	By inhibiting the aromatase enzyme, it reduces the circulating estrogen levels by suppressing its conversion from androgens, predominantly in adipose tissues
GnRHa	Goserelin [21] Leuprolide [22] Triptorelin [23]	3.6 mg SubQ q28 days 3.75 mg IM q28 days 3.75 mg IM q28 days	Chemical castration leading to lower secretion of FSH and LH, thereby suppressing estrogen levels
CDK 4/6 Inhibitor	Abemaciclib [24]	150 mg PO twice daily	Selective inhibition of CDK4 and CDK6, subsequently terminating the cell cycle at the G1 phase by interrupting pRb phosphorylation
Bisphosphonate *	Zoledronic Acid [25] Clodronate [26] Ibandronate [27]	4 mg IV q6 month 1600 mg PO daily 50 mg PO daily	Inhibits osteoclasts by way of apoptosis, and thereby decreases bone resorption and increases bone mineralization

* Dosing, mode of administration and total duration of the bisphosphonates recommended by ASCO guideline [28]: Zoledronic Acid 4 mg IV every 6 months for 3 years or at 4 mg once every 3 months for 2 years. Clodronate 1600 mg PO daily for 2 to 3 years. Ibandronate 50 mg PO daily for 3 years. SERM: Selective Estrogen Receptor Modulator, ER: Estrogen Receptor, PO: Per oral, AI: Aromatase Inhibitor, IV: Intravenous, SubQ: Subcutaneous, IM: Intramuscular, GnRHa: Gonadotropin-releasing hormone agonist, FSH: Follicle stimulating hormone, LH: Luteinizing hormone, CDK 4/6: Cyclin-Dependent Kinase 4/6 inhibitor, pRb: Retinoblastoma protein.

**Table 2:** Summary of key clinical trials, systemic reviews, and meta-analyses that investigated the role of adjuvant endocrine therapy in breast cancer ER+ HER2−.

Study and Treatment	Interval	Recurrence Outcome (95% CI)	Survival Outcome (95% CI)
**Endocrine Therapy 5 Years Standard Duration**			
*5 years of Tamoxifen vs. none* **EBCTCG (2011)** [3] meta-analysis of 20 trials (*n* = 21,457)	Years 0–4 Years 5–9 Years 10–14	Breast cancer recurrence RR 0.53 (0.48–0.57), *p* < 0.0001 RR 0.68 (0.60–0.78), *p* < 0.0001 -	Mortality RR 0.71 (0.62–0.80), *p* < 0.0001 RR 0.66 (0.58–0.75), *p* < 0.0001 RR 0.68 (0.56–0.83), *p* < 0.0001
*5 years of AI vs. 5 years of Tamoxifen* **EBCTCG (2015)** [44] meta-analysis of 9 trials (*n* = 31,920 of postmenopausal women)	Years 0–4 Years 5–9	Breast cancer recurrence RR 0.70 (0.64–0.77), *p* < 0.0001 RR 0.92 (0.83–1.01), *p* = 0.082	Mortality RR 0.79 (0.67–0.92), *p* = 0.002 RR 0.60 (0.50–0.72), *p* < 0.0001
*5 years of Letrozole vs. Tamoxifen* **BIG 1-98 (2018)** [87] randomised control trial (*n* = 8010)	At 8 years At 14 years Years 0–5 Years 5–10 >10 years	DFS HR 0.82 (0.74–0.92), *p* = 0.0002 DFS HR 0.91 (0.81–1.01), *p* = 0.08 Contralateral breast cancer HR 0.62 (0.36–1.09) HR 0.47 (0.23–0.97) HR 1.35 (0.53–3.41)	OS HR 0.79 (0.69–0.90), *p* = 0.0006 OS HR 0.89 (0.77–1.02), *p* = 0.087 -
**Extended Endocrine Therapy Beyond 5 years**			
*5 years of Tamoxifen vs. 10 years of Tamoxifen* **ATLAS (2013)** [58] randomised control trial (*n* = 12,894)	Years 5–9 ≥10 years	Breast cancer recurrence RR 0.90 (0.79–1.02), *p* = 0.10 RR 0.75 [0.62–0.90], *p* = 0.003	Mortality RR 0.97 (0.79–1.18), *p* = 0.74 RR 0.71 (0.58–0.88), *p* = 0.0016
*5 years of Tamoxifen vs. 10 years of Tamoxifen* **aTTom (2013)** [88] randomised control trial (*n* = 6953)	Years 5–6 Years 7–9 >10 years	Breast cancer recurrence RR 0.99 (0.86–1.15) RR 0.84 (0.73–0.95) RR 0.75 (0.66–0.86)	Mortality During 5–9 years: RR 1.03 (0.84–1.27) - > 10 years: RR 0.94 (0.82–1.07)
*5 years of Tamoxifen, followed by 5 years of Letrozole vs. placebo* **NCIC CTG MA.17 (2012)** [89] randomised control trial (*n*= 5187)	At 5 years	DFS HR 0.52 (0.45–0.61), *p* = 0.001	OS HR 0.61 (0.52–0.71), *p* = 0.001
*5 years of ET, followed by 5 years of Letrozole vs. placebo* **NCIC CTG MA.17R (2016)** [90] randomised control trial (*n* = 1918 postmenopausal women)	At 5 years	DFS HR 0.80 (0.63–1.01), *p* = 0.06 DFS rate: 90% (0.88–0.92), with letrozole vs. 88% (0.86–0.90) with placebo	OS HR 0.97 (0.73–1.28), *p* = 0.83 OS rate: 93% (0.92–0.95) with letrozole vs. 94% (0.92–0.95) with placebo
*5 years of Letrozole versus Tamoxifen, and then their sequences* *(2 years of one treatment followed by 3 years of the other)* **BIG 1-98 (2011)** [91] randomised control trial (*n* = 3086)	At 8 years	Letrozole followed by Tamoxifen vs. Letrozole: DFS HR 1.06 (0.91–1.23), *p* = 0.48 Tamoxifen followed by Letrozole vs. Tamoxifen: DFS HR 1.07 (0.92–1.25), *p* = 0.36	Letrozole followed by Tamoxifen vs. Letrozole: OS HR 0.97 (0.80–1.19), *p* = 0.79 Tamoxifen followed by Letrozole vs. Tamoxifen: OS HR 1.10 (0.90–1.33), *p* = 0.36
*5 years of AI (or Tamoxifen for 2–3 years) followed by 5 years of AI* **NSABP B-42 (2019, 2020)** [92,93] randomised control trial (*n* = 3903 of postmenopausal women)	At 7 years At 10 years	DFS HR 0.85 (0.73–0.999), *p* = 0.048 DFS HR 0.84 (0.74–0.96), *p* = 0.011	- OS HR 0.97 (0.82–1.16), *p* = 0.77
5 years of ET*, followed by EET with AI vs. placebo/none **Goldvaser et al. (2017)** [56] systematic review and meta-analysis of 7 trials (*n* = 16,349 patients) *prior duration of ET varied from 2.5–5 years	Median time 12.4 years	Sub-group analysis of LN positive DFS HR 0.72 (0.63–0.83), *p* < 0.001 Sub-group analysis of LN negative DFS HR 0.83 (0.64–1.08), *p* = 0.16	Non-significant DFS among patients with: tumors (cm) >2 vs. ≤ 2 (HR 0.77 vs. HR 0.88) Patients previously treated with chemotherapy: (HR 0.71 vs. HR 0.80), *p* = 0.51
*5 years of ET, followed by EET with AI vs. placebo/none* **Clement et al. (2018)** [57] meta-analysis of 8 trials (*n* = 17,179 postmenopausal women)	At 5 years	DFS OR 1.049 (0.93–1.18), *p* = 0.43 ⋅Contralateral breast cancer: OR 1.094 (0.92–1.30), *p* = 0.31	OS OR 1.033 (0.92–1.15), *p* = 0.56 -
*5 years of ET, followed by EET with AI vs. placebo/none* **Chen et al. (2021)** [64] meta-analysis of 9 trials (*n* = 22,313 postmenopausal women only)	5 to 7–8 years 7–8 to 10 years	Any ET: DFS HR 0.79 (0.69–0.91) Sequential Tamoxifen followed by AI: DFS HR 0.82 (0.71–0.95), DFS HR 0.79 (0.69–0.91)	OS HR 0.90 (0.69–1.17) OS HR 1.02 (0.86–1.20) - OS HR 1.05 (0.90–1.13)
*5 years of ET, followed by EET with Tamoxifen vs. placebo/none* **Al-Mubarak et al. (2014)** [63] meta-analysis of 5 trials (*n* = 21,554)	Median time 9 years	Breast cancer recurrence: OR 0.89 (0.76–1.05), *p* = 0.17 Subgroup of LN-: OR 0.93 (0.76–1.14) Subgroup of LN+: OR 0.76 (0.63–0.92)	No association between EET and all-cause of death, OR 0.99 (0.84–1.16), *p* = 0.88
**Endocrine Therapy in combination with OFS**			
*5 years of OFS + Exemestane vs. OFS vs. Tamoxifen* **SOFT and TEXT (2014, 2016)** [54,94] randomised control trial (*n*= 4690 premenopausal women)	At 5 years	DFS HR 0.72 (0.60–0.85), *p* < 0.001	OS HR 1.14 (0.86–1.51), *p* = 0.03
*5 years of AI + OFS vs. 3–5 years of Tamoxifen + OFS* **EBCTCG (2022)** [55] meta-analysis of 4 trials (*n* = 7030 of premenopausal women)	Years 0–4 Years 5–9 At 10 years	Disease recurrence RR 0.68, (0.55–0.85); *p* < 0.0001 RR 0.98, (0.73–1.33); *p* = 0.89 RR 0.79, (0.69–0.90); *p* = 0.0005	Mortality RR 1.33, (1.00–1.76) RR 0.84, (0.64–1.11) RR 1.01 (0.82–1.24), *p* = 0.94
*5 years of OFS + ET vs. ET alone* **Cochrane (Bui et al., 2020)** [52] systematic review and meta-analysis of 15 trials (*n* = 11,538 premenopausal women)	At 5 years	DFS HR 0.83 (0.77–0.90), *p* < 0.001 Sub-group: OFS + Tamoxifen vs. Tamoxifen DFS HR 0.76 (0.63–0.92), *p* = 0.005	OS HR 0.86 (0.78–0.94), *p* = 0.001 Sub-group: OFS + Tamoxifen vs. Tamoxifen DFS HR 0.74 (0.59–0.93), *p* = 0.009

ET: Endocrine Therapy, EET: Extended Endocrine Therapy AI: Aromatase Inhibitor, OFS: Ovarian Function Suppression, LN: Lymph Node, OS: Overall Survival, DFS: Disease-free Survival, CI: Confidence Interval, HR: Hazard Ratio, OR: Odds Ratio, RR: Risk Ratio.
